# Contribution of primary care to health: an individual level analysis from Tibet, China

**DOI:** 10.1186/s12939-015-0255-y

**Published:** 2015-12-14

**Authors:** Wenhua Wang, Leiyu Shi, Aitian Yin, Zongfu Mao, Elizabeth Maitland, Stephen Nicholas, Xiaoyun Liu

**Affiliations:** 1 School of Public Health, Wuhan University, 115 Donghu Road, Wuhan, 430071 Hubei Province P. R. China; 2 Center for Health Management and Policy, Shandong University, 44 Wenhuaxilu, Jinan, 250012 Shandong Province P. R. China; 3 Johns Hopkins Bloomberg School of Public Health, Johns Hopkins Primary Care Policy Center, 624 North Broadway, Baltimore, MD 21205 USA; 4 School of Management, Australian School of Business, University of New South Wales, Sydney, 2052 NSW Australia; 5 School of Management, Tianjin Normal University, West Bin Shui Avenue, Tianjin, 300074 P. R. China; 6 School of International Business, Beijing Foreign Studies University, 19 North Xisanhuan Avenue, Haidian, Beijing, 100089 P. R. China; 7 Guangdong Research Institute for International Strategies, Guangdong University of Foreign Studies, 2 Baiyun North Avenue, Baiyun, Guangzhou, 510420 Guangdong P. R. China; 8 University of Newcastle, Newcastle, 2308 NSW Australia; 9 China Center for Health Development Studies, Peking University, 38 Xueyuan Road, Haidian District, Beijing, 100191 P. R. China

**Keywords:** Primary care quality, Self-rated health, Primary care assessment tool

## Abstract

**Introduction:**

There have been significant improvements in health outcomes in Tibet, health disparities between Tibet and the rest of China has been greatly reduced. This paper tests whether there was a positive association between good primary care and better health outcomes in Tibet.

**Method:**

A validated Tibetan version of the Primary Care Assessment Tool (PCAT-T) was used to collect data on 1386 patients aged over 18 years old accessing primary care. Self-rated health (SRH) was employed to measure health outcomes. A multiple binary logistic regression model was used to explore the association between primary care quality and self-rated health status after controlling for socio-demographic and lifestyle variables.

**Results:**

This study found that primary care quality had a significant positive association with self-rated health status. Among the nine domains of PCAT-T, family centeredness domain had the highest Odds Ratio (OR = 1.013) with SRH. Patients located in rural area, with higher education levels, without depression, and less frequent drinking were more likely to self-rate as “good health” compared with the reference group.

**Conclusions:**

In Tibet, higher quality primary care was associated with better self-rated health status. Primary care should be much strengthened in future health system reform in Tibet.

## Introduction

The positive relationship between good quality primary health care and beneficial health outcomes has been well-established. Good primary care can lower under-five mortality rates, decrease infant mortality, reduce incidence of low birth weight, decrease inpatient admission, result in fewer outpatient visits, decrease emergency room visits, and lower health care costs [1–11]. Starfield showed that the beneficial effects of primary care were evident not only in industrialized countries, but also in middle and lower income countries [11]. Further, good primary care can reduce racial, ethnic, and income inequality-led health disparities. This relationship is particularly pronounced for the racial and ethnic minorities living at or below poverty level, and good primary care quality was especially beneficial in areas with highest income inequality [12, 13].

Tibet Autonomous Region (TAR) is located in south western China, at an average altitude of more than 4000 m, it covers more than 1.2 million square kilometers, and accounts for one-eighth of China’s geographic area. In 2013, the population in TAR was 3.12 million, of whom more than 90 % are Tibetan people whose native language is Tibetan. The population is predominantly rural; the percentage of urban population is 23.7 % [14].

The health system in Tibet is a primary care based system, mainly comprising both primary care centers and hospital outpatient departments [15, 16]. During the past six decades, Tibet has received funding to improve its health system capacity from many channels, including the local government, the national government, aid from other provinces and international agencies. In 2009, China launched an ambitious health-care reform program that targeted further improvements to the primary health care delivery, including Tibet. Investments in Tibetan health system has achieved falling maternal mortality rates (from 5000/100,000 in 1950s to 154.51/100,000 in 2013), declining infant mortality rates (from 430/1000 to 19.97/1000) and increasing life expectancy (from 35.5 to 68 years) [17]. It is believed the great primary care capacity enhancement contributes directly to the significant health outcome improvement in Tibet [17]. However, there is no empirical evidence to confirm a positive association between good primary care and better health outcome. This paper addresses this lacuna.

Self-rated health (SRH) is a widely used measure by which a person reflects and intuitively summarizes his/her own health state [18]. This indicator has become increasingly popular for assessing health status because of its simplicity and solid well-established links with various health indicators such as mortality, morbidity and biological markers [19–23]. Previous studies have showed that individuals living in states with a higher ratio of primary care physician to population were more likely to report good health, and good primary-care experience, in particular enhanced accessibility and continuity, was positively associated with better self-rated health [12, 13, 24]. These evidences confirm that SRH can be used as a reliable surrogate variable for overall health outcomes.

SRH is based on a respondent’s evaluation of his/her health status on a Likert scale using a global health question (‘In general, how would you rate your health today?’) [18, 25]. There exists a comprehensive measure to Tibetan primary care, the Primary Care Assessment Tool-Tibetan version (PCAT-T), which assesses patient perceived primary care quality [15]. Our study in Tibet adds to the few existing studies to explore the association between primary care and health outcome at individual level in Tibet.

## Methods

### Study design and data collection

The Ethics Committee of Tibet Autonomous Regional Health and Family Planning Commission approved the study. The study was based on face-to-face patient surveys conducted on-site at the sampled primary care providers. A stratified, purposive sampling method was adopted to select 13 representative primary care practices, including two prefecture traditional Tibetan medicine (TTM) hospital outpatient departments, two prefecture western medicine (WM) hospital outpatient departments, three county hospital (CH) outpatient departments, and six township health centers (THC). All patients aged 18 years or older who visited our sampled primary care practices were eligible to participate in the survey. Only patients who reported that the practice they visited was their regular source of primary care were interviewed. Each potential participant was given an explanation of the research purpose and asked for permission to participate in the interview.

For our previous original comparative analysis study, the sample sizes were estimated with reference to other similar studies that showed a sample size of 300 per group was needed for a significance level of 5 % with a power of 90 %. There are four types of primary care practice in our study, so the minimum sample size is 1200. 20 % were added to the estimated sample size in consideration of potential missing data. Therefore, the estimated sample size was 1440 in total. While most of patients approached accepted our invitation to complete this survey, some patients refused, mainly because they needed to travel a long distance back to their home immediately after having the outpatient service. 54 questionnaires were deleted due to missing data, leaving 1386 completed questionnaires. The methodological details were reported in previous studies [15, 16].

### Measures

The PCAT-T consisted of seven multi-item scales and two single-item scales: first contact and continuity, comprehensiveness (medical care), comprehensiveness (social care), first contact (access), coordination, family centeredness, community orientation, same doctor and stableness [16]. We converted Likert scales to scores ranging from 25 to 100 by dividing the Likert scale by 4 and multiplying by 100. Means of item scores in the same scale yielded scale scores, and the primary care total score was the mean of these nine scale scores. The PCAT-T captured patient perceived primary care quality. The certainty as to whether a service was received or not was measured on a 4 point Likert scale, ranging from “1” (“Definitely not”) to “4” (“Definitely”) and the question “In general, how would you rate your health today?” was used to measure patients’ self-rated health status. We coded the five-point Likert scale items “Very good, Good, Neutral, Poor, and Very poor”, to a binary scale as 1 for respondents reporting Very good, good health (labeled good health) and 0 for those reporting Neutral, Poor or Very poor health (labeled poor health), which is consistent with the method employed by comparable previous studies [12, 13, 24]. We also collected a range of individual socio-demographic and lifestyle characteristics known to influence health, including location, gender, age, education, income level, marital status, presence of depression, smoking and drinking habits and physical activity, which were included as control variables in the multiple logistic regression model.

### Statistical analysis

Association between socio-demographic data of the participants and their self-rated health status were analyzed using chi-square tests. Independent sample t-tests were performed to compare primary care assessment scores. Multiple binary logistic regression analysis was conducted to explore the association between primary care quality and self-rated health status after controlling for socio-demographic and life style behavior variables.

## Results

### Self-rated health status by different characteristics

Table 1 shows significant differences in self-rated health status by different socio-economic status and life style behaviors. The good health group tended to locate in rural area, be female, younger, with a higher education, without depression and more likely to be non- smoking and non-drinking, than the poor health group. There were no significant differences in health status among groups in different income level, marital status and exercise frequency.

**Table 1:** Patients’ self-rated health status by different characteristics

Characteristics	Poor health (%) (*n* = 802)	Good health (%) (*n* = 584)	*P*-value^a^
Location			<0.01
Urban	448 (64.7)	244 (35.3)	
Rural	354 (51.0)	340 (49.0)	
Gender			<0.05
Male	394 (61.4)	248 (38.6)	
Female	408 (54.8)	336 (45.2)	
Age			<0.01
≤ 44 years	462 (54.6)	384 (45.4)	
45–59 years	223 (59.0)	155 (41.0)	
≥ 60 years	117 (72.2)	45 (27.8)	
Education			<0.01
Never attend school	337 (67.4)	163 (32.6)	
Primary school	241 (55.9)	190 (44.1)	
Junior high school and above	224 (49.2)	231 (50.8)	
Income^b^ (annual household income)			0.76
≤ 31400RMB	561 (57.6)	413 (42.4)	
> 31400RMB	241 (58.5)	171 (41.5)	
Marital status			0.25
Singled	194 (60.6)	126 (39.4)	
Married	608 (57.0)	458 (43.0)	
Depression			<0.01
Yes	426 (78.2)	119 (21.8)	
No	376 (44.7)	465 (55.3)	
Smoking			<0.01
Current smoker	177 (64.6)	97 (35.4)	
Ex-smoker	100 (64.1)	56 (35.9)	
No smoker	525 (54.9)	431 (45.1)	
Drinking (times per week)			<0.01
≥ 3	199 (70.1)	85 (29.9)	
1–2	145 (58.2)	104 (41.8)	
0	458 (53.7)	395 (46.3)	
Exercise (times per week)			0.10
≥ 3	190 (53.4)	166 (46.6)	
1–2	239 (58.0)	173 (42.0)	
0	373 (60.4)	245 (39.6)	

Poor health: people with low self-rated health (neutral, poor or very poor)

Good health: people with high self-rated health (very good, good)

^a^ *P*-value by chi-square test. Significance level is 0.05

^b^Average annual household income was 31400 RMB among the participants

### Primary care quality by self-rated health status

The *t*-test for the nine domains of primary care quality revealed that first contact and continuity, comprehensiveness (social care), first contact (access), coordination, family centeredness, and community orientation was significantly higher for the good health group than the poor health group. While the good health group reported lower score on the stableness domain. There were no significant differences between the two groups on the comprehensiveness (medical care) domain and same doctor domain (Table 2).

**Table 2:** Comparison of primary care assessment score among adult patients by self-rated health

Scales	Poor health Score Mean(SE)	Good health Score Mean(SE)	*P*-value^a^
First contact and continuity	88.10(0.44)	90.80(0.46)	<0.01
Comprehensiveness (medical care)	79.31(0.61)	80.93(0.81)	0.11
Comprehensiveness (social care)	83.75(0.54)	86.55(0.67)	<0.01
First contact (access)	65.46(0.84)	70.21(1.05)	<0.01
Coordination	81.58(0.64)	84.72(0.78)	<0.01
Family centeredness	85.92(0.48)	87.72(0.53)	<0.05
Community orientation	74.73(0.72)	77.35(0.84)	<0.05
Same doctor	71.15(0.90)	72.04(1.09)	0.53
Stableness	47.62(0.80)	45.00(0.93)	<0.05
Total	79.94(0.36)	82.25(0.42)	<0.01

Higher value indicate a more positive experience

Poor health: people with low self-rated health (neutral, poor or very poor)

Good health: people with high self-rated health (very good, good)

*SE* standard error

^a^ *P*-value by *t* test. Significance level is 0.05

### Association between primary care quality and self-rated health status

Multiple binary logistic regression analysis showed that the primary care assessment total score was positively associated with good health. When the total primary care assessment score increased by 1 point, the probability that the patient rated “good health” increased 2.0 % (Table 3). The scores of all PCAT domains were also associated with good health, except for the first contact and continuity, community orientation, and same doctor. The odd ratio value of family centeredness was the highest (Table 4). For other factors, patients located in rural area, with higher education levels, without presence of depression, less frequent drinking were more likely to rate “good health” compared with the reference group (Table 3).

**Table 3:** Factors associated with good health compared to poor health

Dependent variable: self-rated health	OR (95 % CI)	SE	*P*-value^a^
Primary care assessment total score	1.020(1.008–1.033)	0.006	0.002
Location			
Urban	-		
Rural	1.876(1.415–2.487)	0.144	<0.001
Gender			
Male	-	-	-
Female	1.078(0.821–1.415)	0.139	0.591
Age			
18–44 years	-	-	-
45–59 years	1.023(0.771–1.357)	0.144	0.876
≥ 60 years	0.797(0.522–1.218)	0.216	0.294
Income^b^ (annual household income)			
≤ 31400RMB	-		
> 31400RMB	0.797(0.609–1.043)	0.137	0.098
Education			
Never attend school	-	-	-
Primary school	1.567(1.160–2.116)	0.153	0.003
Junior high school and above	2.976(2.111–4.195)	0.175	<0.001
Marital status			
Singled	-		
Married	1.319(0.988–1.762)	0.148	0.060
Depression			
Yes	-		
No	3.961(3.057–5.130)	0.132	<0.001
Smoking			
Current smoker	-	-	-
Ex-smoker	1.129(0.712–1.792)	0.236	0.606
No smoker	1.166(0.818–1.662)	0.181	0.396
Drinking (times per week)			
≥ 3	-	-	-
1–2	1.762(1.181–2.630)	0.204	0.006
0	2.039(1.440–2.887)	0.177	<0.001
Exercise (times per week)			
≥ 3	-	-	-
1–2	1.101(0.801–1.515)	0.163	0.553
0	0.976(0.723–1.318)	0.153	0.876

Poor health: people with low self-rated health (neutral, poor or very poor)

Good health: people with high self-rated health (very good, good)

*SE* standard error, *OR* odds ratio, *CI* confidence interval

^a^ *P*-value by multiple logistic regression analysis. Significance level is 0.05

^b^Average annual household income was 31400 RMB among the participants

**Table 4:** Domain scores associated with good health compared to poor health

Domains	OR (95 % CI)	SE	*P*-value^a^
First contact and continuity	1.011(1.000–1.022)	0.006	0.054
Comprehensiveness (medical care)	1.009(1.002–1.015)	0.003	0.010
Comprehensiveness (social care)	1.010(1.002–1.018)	0.004	0.011
First contact (access)	1.008(1.003–1.013)	0.003	0.003
Coordination	1.010(1.003–1.017)	0.003	0.003
Family centeredness	1.013(1.004–1.023)	0.005	0.005
Community orientation	1.001(0.995–1.007)	0.003	0.763
Same doctor	1.003(0.998–1.007)	0.002	0.228
Stableness	0.993(0.988–0.999)	0.003	0.016

Poor health: people with low self-rated health (neutral, poor or very poor)

Good health: people with high self-rated health (very good, good)

All models were adjusted for location, gender, age, education, income level, marital status, depression, smoking, drinking habits and exercise factors

*SE* standard error, *OR* odds ratio, *CI* confidence interval

^a^ *P*-value by multiple logistic regression analysis. Significance level is 0.05

## Discussion

This study examined the association between patient perceived primary care quality and self-rated health status in an autonomous region in China. Our results revealed that the primary care assessment total score was positively associated with self-rated health status. The findings are consistent with similar U.S. and Korean studies [12, 13, 24]. For each domain, the family centeredness domain score had the highest Odds Ratio. This means family centeredness played the most important role in improving health outcome in Tibetan area. Family centeredness refers to recognition of family factors related to genesis and management of illness. The policy implication is that family characteristic should be more considered in primary care policy making. This finding is consistent with a currently ongoing national pilot program, which requires all family physicians at primary care practices should establish a service contract with families and residents in their service community [26].

For other factors, patients with higher education level had higher probability to report healthy, which is consistent with Shi’s study [13]. Patients living in rural area reported better health status than those living in urban area, this could be explained by the fact that people living in rural areas have been keeping some good traditional habits, such as religious activities, and had a more harmonious community culture, which led to positive self-reported health status.

Among life style behaviors, drinking has a significant effect on self-rated health. Our study revealed the same results as previous studies that found that heavier drinkers reported lower health status [27–30]. In Tibet, drinking beer is very popular, and many people engage in prolonged periods of beer drinking. Lack of rest and heavy alcohol use helps explain lower self-reported health status.

Our study has several limitations. Both a unique advantage and a limitation, a self-reported health survey was used to measure primary care quality. Some aspects of technical quality cannot be assessed from patients’ perceptions, because of their limited clinical knowledge. Recall bias may also intervene. Despite these issues, patients’ self-reports are widely accepted as an important method of measuring health care quality and health care performance especially when alternative health care performance measures are not available [31]. Second, there were variables, such as health history, social capital, income inequality, where no data were available, but which might have influenced self-rated health status. These types of data might merit future study. Third, due to the cross-sectional nature of this study, our study only explored the association between primary care quality and self-rated health, and it is difficult to identify a causal relationship between primary care quality and self-rated health.

## Conclusion

In Tibet, where little empirical evidence exists to measure the impact of primary care on health performance, SRH survey data provide an alternative measure of health outcomes. Our study shows that primary care total score as well as specific domain score, is positively associated with better self-rated health outcomes. This study suggests that further primary care capacity building should pay more attention to the field of family centeredness area in Tibet.

## References

[1] Starfield B (1992). Primary care: concept, evaluation, and policy.

[2] Shi L (1992). The relationship between primary care and life chances. J Health Care Poor Underserved.

[3] Shi L, Macinko J, Starfield B, Xu J, Regan J, Politzer R (2004). Primary care, infant mortality, and low birth weight in the states of the USA. J Epidemiol Community Health.

[4] Macinko J, Starfield B, Erinosho T (2009). The impact of primary health care on population health in low- and middle-income countries. J Ambul Care Manage.

[5] Bindman AB, Grumbach L, Osmond D (1996). Primary care and receipt of preventive services. J Gen Intern Med.

[6] Starfield B, Shi L, Macinko J (2005). Contribution of primary care to health systems and health. Milbank Q.

[7] Rohde J, Cousens S, Chopra M, Tangcharoensathien V, Black R, Bhutta ZA (2008). 30 years after Alma-Ata: has primary health care worked in countries?. Lancet.

[8] Kruk ME, Porignon D, Rockers PC, Van Lerberghe W (2010). The contribution of primary care to health and health systems in low- and middle-income countries: a critical review of major primary care initiatives. Soc Sci Med.

[9] Kringos DS, Boerma WG, Hutchinson A, van der Zee J, Groenewegen PP (2010). The breadth of primary care: a systematic literature review of its core dimensions. BMC Health Serv Res.

[10] Friedberg MW, Hussey PS, Schneider EC (2010). Primary care: a critical review of the evidence on quality and costs of health care. Health Aff.

[11] Starfield B (2012). Primary care: an increasingly important contributor to effectiveness, equity, and efficiency of health services. SESPAS report 2012. Gac Sanit.

[12] Shi L, Starfield B (2000). Primary care, income inequality, and self-rated health in the United States: a mixed-level analysis. Int J Health Serv.

[13] Shi L, Starfield B, Politzer R, Regan J (2002). Primary care, self-rated health, and reductions in social disparities in health. Health Serv Res.

[14] Tibet Autonomous Regional Statistics Bureau (2014). Tibet statistical yearbook 2014.

[15] Wang W, Shi L, Yin A, Lai Y, Maitland E, Nicholas S (2014). Development and validation of the Tibetan primary care assessment tool. Biomed Res Int.

[16] Wang W, Shi L, Yin A, Mao Z, Maitland E, Nicholas S (2015). Primary care quality among different health care structures in Tibet, China. Biomed Res Int.

[17] Maternal Mortality Rate in Tibet Autonomous Region. 2014. http://news.xinhuanet.com/2014-04/06/c_1110125362.htm. Accessed 6 Apr 2014.

[18] Hirve S, Juvekar S, Sambhudas S, Lele P, Blomstedt Y, Wall S (2012). Does self-rated health predict death in adults aged 50 years and above in India? Evidence from a rural population under health and demographic surveillance. Int J Epidemiol.

[19] Jia Y, Gao J, Dai J, Zheng P, Wu X, Li G (2014). Difference of the associations between self-rated health and demographic characteristics, lifestyle, and psychosocial work environment between two types of Chinese worksite. BMC Public Health.

[20] Abdulrahim S, El Asmar K (2012). Is self-rated health a valid measure to use in social inequities and health research? Evidence from the PAPFAM women’s data in six Arab countries. Int J Equity Health.

[21] Idler EL, Benyamini Y (1997). Self-rated health and mortality: a review of twenty-seven community studies. J Health Soc Behav.

[22] Jylha M, Volpato S, Guralnik JM (2006). Self-rated health showed a graded association with frequently used biomarkers in a large population sample. J Clin Epidemiol.

[23] Kaplan GA, Pamuk ER, Lynch JW, Cohen RD, Balfour JL (1996). Inequality in income and mortality in the United States: analysis of mortality and potential pathways. BMJ.

[24] Sung NJ, Markuns JF, Park KH, Kim K, Lee H, Lee JH (2013). Higher quality primary care is associated with good self-rated health status. Fam Pract.

[25] Jeroen KVG, George G (2012). A single- vs. multi-item self-rated health status measure: a 21-country study. Open Public Health J.

[26] National Health and Family Planning Commission (2013). Guidance for rural doctor contract service pilot.

[27] Tsai J, Ford ES, Li C, Pearson WS, Zhao G (2010). Binge drinking and suboptimal self-rated health among adult drinkers. Alcohol Clin Exp Res.

[28] Valencia-Martín JL, Galán I, Rodríguez-Artalejo F (2009). Alcohol and self-rated health in a Mediterranean country: the role of average volume, drinking pattern, and alcohol dependence. Alcohol Clin Exp Res.

[29] Saarni S, Joutsenniemi K, Koskinen S, Suvisaari J, Pirkola S, Sintonen H (2008). Alcohol consumption, abstaining, health utility and quality of life: a general population survey in Finland. Alcohol.

[30] Okosun IS, Seale JP, Daniel JB, Eriksen MP (2005). Poor health is associated with episodic heavy alcohol use: evidence from a National Survey. Public Health.

[31] Martin R (2012). Measuring and improving patient experience in primary care. Prim Health Care Res Dev.

